# Depiction of mosaic perfusion in chronic thromboembolic pulmonary hypertension (CTEPH) on C-arm computed tomography compared to computed tomography pulmonary angiogram (CTPA)

**DOI:** 10.1038/s41598-021-99658-2

**Published:** 2021-10-08

**Authors:** Sabine K. Maschke, Thomas Werncke, Cornelia L. A. Dewald, Lena S. Becker, Timo C. Meine, Karen M. Olsson, Marius M. Hoeper, Frank K. Wacker, Bernhard C. Meyer, Jan B. Hinrichs

**Affiliations:** 1 Department of Diagnostic and Interventional Radiology, Member of the German Center for Lung Research (DZL), Hannover Medical School, Carl-Neuberg-Str. 1, 30625 Hannover, Germany; 2 Clinic for Pneumology, Member of the German Center for Lung Research (DZL), Hannover Medical School, Hannover, Germany

**Keywords:** Medical research, Biomarkers, Diagnostic markers, Interventional cardiology

## Abstract

To evaluate mosaic perfusion patterns and vascular lesions in patients with chronic thromboembolic pulmonary hypertension (CTEPH) using C-Arm computed tomography (CACT) compared to computed tomography pulmonary angiography (CTPA). We included 41 patients (18 female; mean age 59.9 ± 18.3 years) with confirmed CTEPH who underwent CACT and CTPA within 21 days (average 5.3 ± 5.2). Two readers (R1; R2) independently evaluated datasets from both imaging techniques for mosaic perfusion patterns and presence of CTEPH-typical vascular lesions. The number of pulmonary arterial segments with typical findings was evaluated and the percentage of affected segments was calculated and categorized: < 25%; 25–49%; 50–75%; < 75% of all pulmonary arterial segments affected by thromboembolic vascular lesions. Inter-observer agreement was calculated for both modalities using the intraclass-correlation-coefficient (ICC). Based on consensus reading the inter-modality agreement (CACT_cons_ vs. CTPA_cons_) was calculated using the ICC. Inter-observer agreement was excellent for central vascular lesions (ICC > 0.87) and the percentage of affected segments (ICC > 0.76) and good for the perceptibility of mosaic perfusion (ICC > 0.6) and attribution of the pattern of mosaic perfusion (ICC > 0.6) for both readers on CACT and CTPA. Inter-modality agreement was excellent for the perceptibility of mosaic perfusion (ICC = 1), the present perfusion pattern (ICC = 1) and central vascular lesions (ICC = 1). However, inter-modality agreement for the percentage of affected segments was fair (ICC = 0.50), with a greater proportion of identified affected segments on CACT_cons_. CACT demonstrates a high agreement with CTPA regarding the detection of mosaic perfusion. CACT detects a higher number of peripheral vascular lesions compared to CTPA.

## Introduction

Chronic thromboembolic pulmonary hypertension (CTEPH) presents one out of five types of pulmonary hypertension^1^. In CTEPH, incomplete resolution and consecutive organization of acute pulmonary emboli causes chronic thromboembolic vascular obstruction. Up to 4% of patients who survived one or more episodes of pulmonary embolism develop CTEPH^2^. However, diagnosis of CTEPH and its differentiation to other types of pulmonary hypertension can be challenging^3^. Besides typical findings on V/Q scintigraphy and in right heart catheterization, the diagnosis of CTEPH requires typical vascular findings in a suitable imaging modality^4^. Computed tomography pulmonary angiography (CTPA) is an established imaging modality to diagnose CTEPH by visualizing CTEPH-typical vascular findings^4^. Unfortunately, a negative CTPA cannot definitively rule out CTEPH as lesions in small, peripheral branches often stay unnoticed^4–6^. However, mosaic perfusion detected on CTPA, is a frequent secondary diagnostic parameter in patients suffering from CTEPH, even in the absence of vascular lesions, reflecting the severity of perfusion changes due to chronic pulmonary embolism^3,7,8^.

Therefore, detection of mosaic perfusion on CTPA in patients with known pulmonary hypertension should trigger further diagnostic work-up, typically including digital subtraction angiography (DSA)^1,4^. C-Arm computed tomography (CACT) of the pulmonary arteries is an alternative 3D imaging modality that combines imaging features typical for DSA and CTPA. It can easily be acquired during the same session as DSA and offers images with a high spatial resolution that can lead to a more comprehensive imaging work-up of patients with suspected CTEPH^9^. As described previously, CACT provides a more detailed depiction of distal thromboembolic lesions when compared to CTPA^9^. The addition of CACT to the DSA workup improves both, diagnosis and detection of CTEPH-typical vascular lesions which is of decisive importance for the determination of the most suitable treatment option^3,9–11^. However, the perceptibility of mosaic perfusion on CACT has not been evaluated yet, even though it might offer a more comprehensive and functional approach of CTEPH-induced pathologies than detection of vascular lesions alone.

We aimed to evaluate the perceptibility of patterns of mosaic perfusion and peripheral vascular lesions typical for CTEPH on CACT in comparison to CTPA and to determine its diagnostic value for detection of perfusion inhomogeneities of the lung parenchyma.

## Material and methods

Our local ethics committee (Ethics committee Hannover Medical School, Hannover, Germany) approved this retrospective study. Informed consent was waived by the same committee. All experiments were performed in accordance with relevant guidelines and regulations. Between June 2013 and July 2020 420 patients (204 female (48.6%), 216 male (51.4%); mean age 59.9 ± 18.3 years) underwent our standardized diagnostic work-up for suspected CTEPH as described elsewhere, including DSA and CACT in our angiographic suit ^12^. For 54 patients (12,9%) an additional CTPA was available for review in our picture archiving and communication system. Out of these 54 patients, we included 41 (18 female (44%), 23 male (56%)) who met our predefined criteria: 1. confirmed CTEPH according to current guidelines (mean pulmonary arterial pressure (mPAP) 43.5 ± 11.6 mmHg)^4^. 2. additional CTPA, (a) meeting the minimally required image quality criteria according to the American College of Radiology recommendations for computed tomography for the evaluation of pulmonary embolism^13^ and (b) performed within a maximum of 21 days (5.3 ± 5.2 days) to rule out acute pulmonary embolism as the cause of sudden, newly occurred or significantly worsened shortness of breath. However, CTPA could rule out acute pulmonary embolism or pulmonary consolidations.

Parts of the presented study population has been included previously in other studies. However, the perceptibility of parenchymal lung pathologies on CACT compared to CTPA was not the aim of the previous studies. For patient demographics see Table 1.

**Table 1:** Patient characteristics.

Age	59.9 ± 18.4
Sex (n, %)	18 female (44), 23 male (56)
mPAP (mmHg)	43.5 ± 11.6 mmHg
**Treatment**	
Balloon pulmonary angioplasty	8 (19.5)
Pulmonary endartherectomy	33 (80.5)

### CACT

All procedures were performed on a monoplane, ceiling-mounted angiographic system (Artis Q, Siemens Healthineers, Forchheim, Germany) or on a monoplane, robotic-arm-mounted angiographic system (Artis pheno, Siemens Healthineers, Forchheim, Germany). A 5 F pigtail catheter (Cordis, Waterloo, Belgium) was placed in the pulmonary trunk. CACT was obtained using the manufacturer’s preset during contrast injection (total injected volume 70 mL, comprising 49 mL Iomeprol 300 mgI/mL and 21 mL saline, effective iodine concentration 210 mgI/mL, flow rate 8 mL/s) within a single modest breath hold as described previously^12^. In order to optimize the anatomical coverage, the field of view was centered on the central pulmonary arteries under fluoroscopic control.

### CTPA

We included 41 contrast-enhanced multidetector CTPA. 32 (78.0%) were performed at our institution and 9 (22.0%) performed outside of our hospital. All images were available for review in our picture archiving and communication system.

To address potential heterogeneity of the images, we defined minimally required image quality criteria according to the American College of Radiology recommendations for computed tomography for the evaluation of pulmonary embolism^13^. In brief: CTPA were performed on a multidetector-row CT to detect pulmonary embolism, using automated bolus tracking in the main pulmonary artery and automated contrast material injection with a flow rate of 3 mL per second, with a slice thickness of ≤ 1.5 mm and acquired within a maximum of 21 days prior to or past CACT. The mean slice thickness was 1.26 ± 0.31 mm, the mean period between CACT and CTPA acquisition measured 5.3 ± 5.2 days. 11 CTPA (26.8%) were perfomed after previous CACT, 30 CTPA (73.2%) afterwards.

### Image analysis

**Figure 1:**
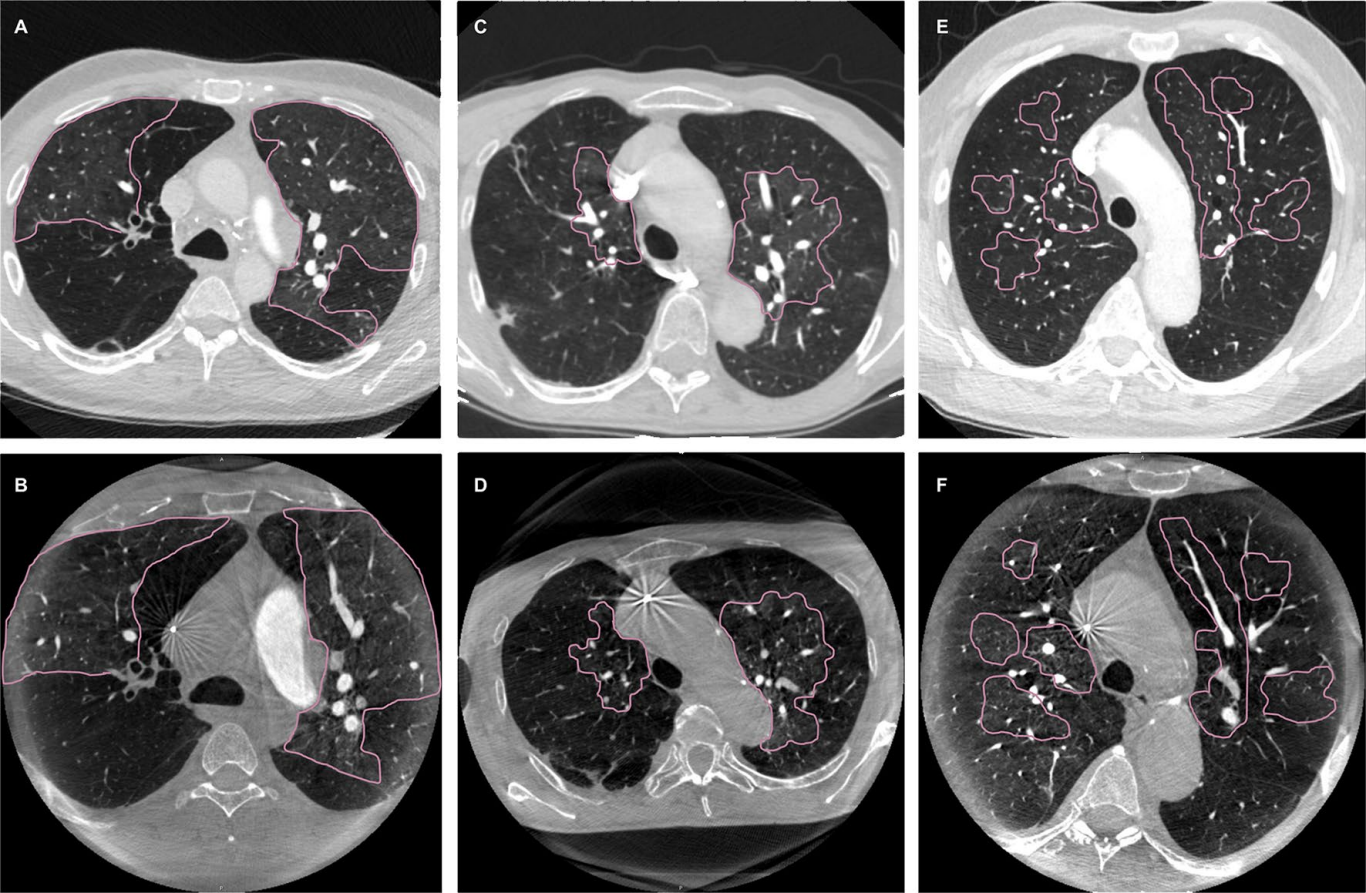
Patterns of mosaic perfusion on CT and CACT. (**A**, **B**) pattern 1, sharply demarcated segmental and/or subsegmental areas of hypo- and hyperattenuation with well-defined borders corresponding to the anatomic unit of the secondary pulmonary lobule on CT (**A**) and CACT (**B**); (**C**, **D**) pattern 2, perihiliar hyperattenuating areas with peripheral perfusion defects on CT (**C**) and CACT (**D**); (**E**, **F**) pattern 3, diffuse heterogenity of lung attenuation with patchy low-attenuating areas located more centrally within in the secondary lobule and intermixed with areas of normal or increased attenuation on CT (**E**) and CACT (**F**).

During two separate sessions and in random order of patients, two readers with 5 and 9 years (R1: S.K.M.; R2: J.B.H.) of clinical experience in cardiovascular and interventional imaging independently reviewed anonymized images of both modalities. The CACT images were analyzed during a first session and the CTPA images during a second session as described previously^12^. CACT and CTPA were reviewed regarding three categories: First, the presence of mosaic perfusion was assessed. If present, mosaic perfusion was divided into three patterns according to Grosse et al.: pattern 1, sharply demarcated segmental and/or subsegmental areas of hypo- and hyperattenuation with well-defined borders corresponding to the anatomic unit of the secondary pulmonary lobule; pattern 2, perihiliar hyperattenuating areas with peripheral perfusion defects; and pattern 3, diffuse heterogenity of lung attenuation with patchy low-attenuating areas located more centrally within in the secondary lobule and intermixed with areas of normal or increased attenuation^14^ (Fig. 1). Second, thromboembolic vascular findings typical for CTEPH were assessed: stenosis, wall-adherent thrombi, intraluminal structures, abrupt narrowing, complete obstruction on central (central pulmonary artery to segmental pulmonary arteries) or peripheral (sub-segmental) levels of the pulmonary vascular tree. Third, the number of pulmonary arterial segments with findings typical for CTEPH was evaluated. Accordingly, the percentage of affected segments was calculated and categorized as follows: < 25%; 25–49%; 50–75%; < 75% of all pulmonary arterial segments are affected by thromboembolic pathologies typical for CTEPH.

### Data evaluation

Inter-observer agreement for CACT and CTPA was calculated for all three categories. Subsequently, a CACT consensus (CACT_cons_) and a CTPA consensus (CTPA_cons_) were created based on the documented findings of both readers. If there was disagreement, the images were re-assessed by both readers together in order to reach a consensus. Finally, inter-modality agreement for CACT_cons_ and CTPA_cons_ was calculated.

### Statistical analysis

Descriptive statistical analyses of the patient demographics were calculated (mean value ± standard deviation). Inter-observer and inter-modality agreement of all categories were determined by using the intraclass-correlation-coefficient (ICC). The following classification was used for interpretation: poor (< 0.40); fair (0.4–0.59); good (0.6–0.74); excellent (≥ 0.75)^15^. Statistical analysis was conducted with R (version 3.6.1, http://www.r-project.org with package “IRR” version 0.84.1).

## Results

### Inter-observer agreement

Inter-observer agreement between R1 and R2 was excellent for the perceptibility of central vascular lesions (CTPA: ICC = 0.88, CACT: ICC = 0.88) and the percentage of affected segments (CTPA: ICC = 0.92, CACT: ICC = 0.77). Inter-observer agreement was good for the perceptibility of mosaic perfusion (CTPA: ICC = 0.66, CACT: ICC = 0.66) and attribution of the pattern of mosaic perfusion (CTPA: ICC = 0.62, CACT: ICC = 0.61; also refer to Table 2).

**Table 2:** Inter-observer agreement between Reader 1 (R1) and Reader 2 (R2).

	R1:R2 CTPA	R1:R2 CACT
Perceptibility of mosaic perfusion (ICC)	0.66 (0.44–0.80)	0.66 (0.45–0.80)
Perceptibility of central vascular lesions (ICC)	0.88 (0.78–0.93)	0.88 (0.78–0.93)
Percentage of affected segments (ICC)	0.92 (0.84–0.95)	0.77 (0.61–0.87)
Attribution of the pattern of mosaic perfusion (ICC)	0.61 (0.38–0.78)	0.61 (0.37–0.77)

### Inter-modality agreement

**Figure 2:**
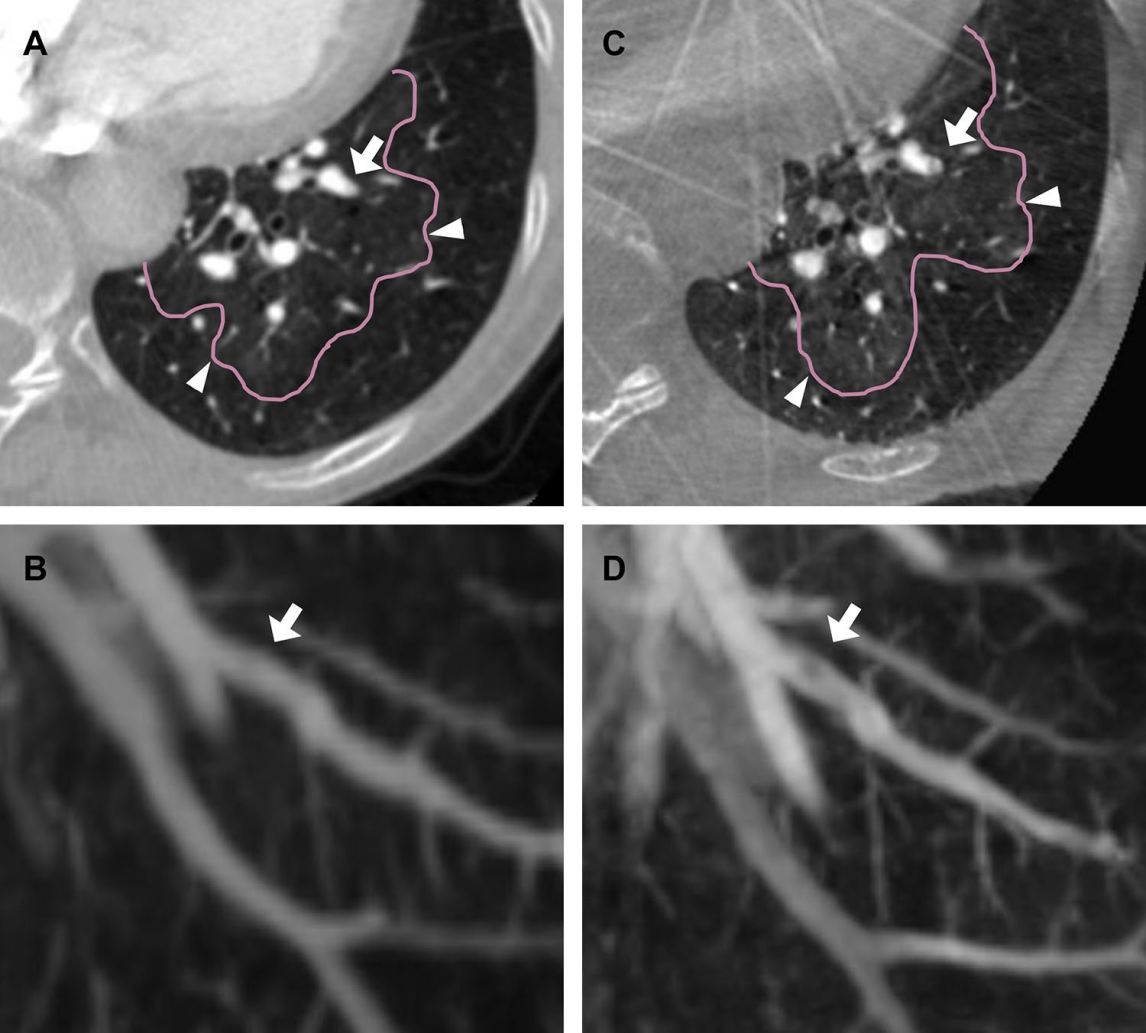
Depiction of peripheral intravascular lesions on CT and CACT. (**A**, **C**) axial multiplanal refomation of the left lower lobe shows mosaic perfusion (arrow heads) on CTPA (**A**) and CACT (**C**). (**B**, **D**) corresponding 20 mm maximal intensity projection of CTPA (**B**) and CACT (**D**) reveals an intravscular band in pulmonary artery segment VIII (arrow) as a typical finding for CTEPH on CACT (**D**) but not on CTPA (**B**).

After conducting a consensus reading, CACT_cons_ and CTPA_cons_ were used to calculate inter-modality agreement. Inter-modality agreement between CACT_cons_ and CTPA_cons_ for the perceptibility of mosaic perfusion was excellent (ICC = 1): with both modalities, mosaic perfusion was present in 40 patients (97.6%), in 1 patient (2.4%) it was not. The inter-modality agreement for the attribution of the pattern of mosaic perfusion was also excellent (ICC = 1): on both modalities, pattern 1 occurred in 28 patients (68.3%), pattern 2 in 7 patients (17.1%) and pattern 3 in 5 patients (12.2%; Table 3). Furthermore, inter-modality agreement for the perceptibility of central vascular lesions was also excellent (ICC = 1): on both modalities, 29 patients (70.7%) showed central lesions, 12 (29.3%) did not (Fig. 2). However, inter-modality agreement for the percentage of affected segments was fair (ICC = 0.50) with an overall higher percentage of pulmonary arterial segments displaying intravascular lesions typical for CTEPH on CACT compared to CTPA (*p*-Value < 0.001). On CACT no patient (0%) showed less than 25% of affected segments, 1 patient (2.4%) 25–49%, 5 patients (12.2%) 50–75% and 35 patients (85.4%) more than 75%. Though, on CTPA 2 patients (4.9%) showed less than 25% of segments affected, 3 patients (7.3%) 25–49%, 16 patients (39.0%) 50–75% and 20 patients (48.8%) more than 75%.

**Table 3:** Inter-modality agreement between CTPA_cons_ and CACT_cons._

	CTPA_cons_:CACT_cons_
Perceptibility of mosaic perfusion (ICC)	1 (1.0–1.0)
Perceptibility of central vascular lesions (ICC)	1 (1.0–1.0)
Percentage of affected segments (ICC)	0.5 (0.01–0.76) ***
Attribution of the pattern of mosaic perfusion (ICC)	1 (1.0–1.0)

***p < 0.001, values are given in mean with 95% confidence interval, as the ICC for any other parameter is 1, no p-Values are given.

## Discussion

CACT and CTPA yielded excellent agreement for the diagnosis of mosaic perfusion and the detection of different perfusion patterns. Furthermore, the number of pulmonary arterial segments with intravascular lesions typical for CTEPH was higher with CACT compared to CTPA. Although there was no gold standard, the higher number of CTEPH-typical findings detected by CACT in combination with the visualization of mosaic perfusion emphasizes the added value of CACT in the diagnostic work-up of patients with CTEPH.

The depiction of peripheral web-like stenoses, intraluminal bands and total occlusions in segmental and sub-segmental pulmonary arteries is of high significance in the diagnostic work-up of patients with suspected CTEPH, as this might be a decisive criterion for both, final diagnosis and therapeutic decision^4,16^. However, especially in patients with predominantly distal thromboembolic lesions, secondary signs are a valuable diagnostic criteria for CTEPH when using CTPA as initial imaging modality^4^. It has been shown that segmental vessel size disparity, mosaic perfusion, enlarged bronchial arteries and bronchial dilatation can be used to reliably distinguish CTEPH from other clinical groups of pulmonary hypertension^3^. Among these, mosaic perfusion, as a sign of perfusion irregularities, is an important criterion to confirm pulmonary hypertension caused by thromboembolic vascular alterations^3^. Therefore, mosaic perfusion on CTPA, even in the absence of vascular findings, should trigger a dedicated diagnostic work-up which usually includes DSA as the imaging modality of choice.

In this context, CACT is a reasonable addition in the diagnostic work-up of patients with suspected CTEPH. As the development of digital, flat-panel detector angiographic systems with high frame rates enabled three dimensional tomographic reconstructions by combining valuable imaging characteristics from both, DSA and CTPA, CACT can easily be combined with acquisition of DSA during one session in the angiographic suite^17–19^. Local administration of contrast agent in the main pulmonary artery can compensate the, compared to multi detector CT, slightly inferior contrast and temporal resolution, as it results in an exclusive and strong enhancement of the pulmonary arteries, enabling an excellent arterial and venous phase separation, facilitating the identification of distal thromboembolic lesions^1,9,10,17–19^. Altogether offering a three dimensional data set with high spatial resolution of peripheral, subsegmental intravascular lesions as well as additional information regarding the vascular wall, adjacent bronchi and the surrounding tissue^1,9,10^. Previous studies confirmed that CACT is superior in visualizing intravascular lesions in the subsegmental pulmonary arteries compared to conventional DSA and CTPA^10,11^. This is reflected in our study by the higher number of affected segments recognized on CACT. In addition, mosaic perfusion, a marker for regional perfusion differences can also be detected by CACT which has not been evaluated so far. This might be of value to assess the extent and characteristics of microperfusion defects.

However, when evaluating its applicabiltity and possible benefits, it needs to be considered that CACT causes additional radiation exposure and additional need for contrast media. The effective radiation dose of thoracic CACT is comparable to that of multi detector CT^10^. The average amount of contrast media required for the assessment of the pulmonary arteries using newest CT scanners is 80 mL and thus, approximately twice as much as is usually used in CACT^12,20,21^. This has to be balanced out with the advantages of CACT, which facilitates the detection of mosaic perfusion and the distinction of different patterns equivalent to CTPA combined with a superior depiction of peripheral vascular lesions^12^.

Sherrick et al. showed that mosaic attenuation due to perfusion irregularities has a significantly higher frequency in patients with pulmonary hypertension due to vascular disease. However, the authors did not clarify how many patients with CTEPH showed mosaic perfusion, as they did not discriminate chronic or acute pulmonary embolism as cause for pulmonary hypertension^8^. Furthermore, Bergin et al. described mosaic perfusion in combination with segmental vessel size disparity to be highly specific for CTEPH. However, the true prevalence of mosaic perfusion in the study population remains unclear as the authors did not distinguish between mosaic attenuation due to infiltrative lung or small airways disease and mosaic attenuation due to occlusive vascular disease^7^.

In 2017, Grosse et al. evaluated prevalence of vascular and parenchymal lung pathologies on CTPA in patients with different causes of pulmonary hypertension to determine secondary diagnostic signs for differentiating CTEPH from non-thromboembolic pulmonary hypertension^3^. Both, vascular and parenchymal CT findings were useful to differentiate between CTEPH and other forms of pulmonary hypertension. Furthermore, they found that a combination of mosaic perfusion and segmental vessel size disparity was highly specific for CTEPH, whereas mosaic perfusion alone also frequently appeared in pulmonary arterial hypertension^3^.

In an additional study, Grosse et al. evaluated CT imaging findings in patients newly diagnosed with CTEPH. They surveyed the frequency of mosaic perfusion and distinguished between different patterns of mosaic perfusion and their incidence. Mosaic perfusion occurred with a frequency of 92.1%. Accordingly, we observed mosaic perfusion in 97.6% of patients. Perfusion pattern 1 also was the most frequent (68.3%) in our study, followed by pattern 2 (17.1%) and pattern 3 (12.2%). Furthermore, Grosse et al. reported a significantly higher frequency of mosaic perfusion in patients with peripheral pulmonary embolism in comparison to patients with exclusively central thromboembolic lesions^14^. In our study, all patients showed peripheral thromboembolic lesions. This finding might explain the even higher incidence of mosaic perfusion in our study compared to the results of Grosse et al.

In patients with CTEPH, areas of hyperattenuation are most likely caused by local hyperperfusion^7,16,22^: it has been shown that systemic blood flow to the lung increases significantly after acute pulmonary embolism and increases even further in patients with CTEPH^5,6,23,24^. The extent of systemic collateral perfusion is a well-known, valid prognostic factor for postsurgical outcome after pulmonary endarterectomy^25^. The occurrence and characteristics of mosaic perfusion, as they indirectly reflect the severity of perfusion impairment, can be used to estimate the dimension of systemic collateral perfusion, hence possibly serving as a tool to estimate the therapeutic benefit. However, Oikonomou et al. did not find a connection between the presence of mosaic perfusion and postoperative outcome after pulmonary endartherectomy (PEA)^25^. Though, the outcome, as measured by decrease of mean pulmonary arterial pressure, was estimated shortly after the surgery, when removing the peri-operative Swan Ganz catheter and transferring the patients from Intensive Care Unit^25^. It is possible, that microvascular changes prior to therapy might take longer to show significant improvement. Therefore, an additional follow up at a later date seems reasonable.

Therefore, its reliable depiction on CACT once more emphasizes the value of CACT in the diagnostic work-up of patients with suspected CTEPH: CACT not only enables improved detection of vascular lesions compared to CTPA or DSA but additionally also offers a more functional approach of CTEPH-induced pathologies than detection of vascular lesions alone, something unachievable with DSA alone.

Our study has several limitations. First, CACT and CTPA have not been acquired at the same day. However, patients did not undergo therapeutic interventions during the interval between both examinations and we did not find differences regarding the presence and pattern of mosaic perfusion on both modalities. Second, CTPA has been acquired on different scanners and with slightly different protocols. Though, we defined the minimum required image quality criteria to ensure comparability. A correlation of histologic probes with the peripheral vascular leasons only detected on CACT and possibly treatable with BPA is impossible as no thromboembolic material is removed during the procedure. Therefore, a gold standard and terminal proof cannot be carried out. Furthermore, our study was done at a single institution with a relatively small number of patients.

## Conclusion

CACT demonstrates a high agreement with CTPA regarding the detection of mosaic perfusion and the distinction of different mosaic perfusion patterns in patients with CTEPH. CACT detects a higher number of peripheral vascular lesions compared to CTPA, indicating the value of additional CACT in the diagnostic work-up of patients with CTEPH eligible for surgery or balloon pulmonary angioplasty (BPA).

## Abbreviations

BPA: Balloon Pulmonary Angioplasty
CACT: C-Arm computed tomography
CTEPH: Chronic thromboembolic pulmonary hypertension
CTPA: Computed tomography pulmonary angiography
DSA: Digital subtraction angiography
ICC: Intraclass-correlation-coefficient
mPAP: Mean pulmonary arterial pressure
PEA: Pulmonary endartherectomy
